# Non-Contact Infrared Thermometers and Thermal Scanners for Human Body Temperature Monitoring: A Systematic Review

**DOI:** 10.3390/s23177439

**Published:** 2023-08-26

**Authors:** Yuanzhe Zhao, Jeroen H. M. Bergmann

**Affiliations:** Department of Engineering Science, University of Oxford, Parks Road, Oxford OX1 3PJ, UK; yuanzhe.zhao@stx.ox.ac.uk

**Keywords:** body temperature, infrared thermometer, thermal scanner, infrared camera, non-contact thermometer

## Abstract

In recent years, non-contact infrared thermometers (NCITs) and infrared thermography (IRT) have gained prominence as convenient, non-invasive tools for human body temperature measurement. Despite their widespread adoption in a range of settings, there remain questions about their accuracy under varying conditions. This systematic review sought to critically evaluate the performance of NCITs and IRT in body temperature monitoring, synthesizing evidence from a total of 72 unique settings from 32 studies. The studies incorporated in our review ranged from climate-controlled room investigations to clinical applications. Our primary findings showed that NCITs and IRT can provide accurate and reliable body temperature measurements in specific settings and conditions. We revealed that while both NCITs and IRT displayed a consistent positive correlation with conventional, contact-based temperature measurement tools, NCITs demonstrated slightly superior accuracy over IRT. A total of 29 of 50 settings from NCIT studies and 4 of 22 settings from IRT studies achieved accuracy levels within a range of ±0.3 °C. Furthermore, we found that several factors influenced the performance of these devices. These included the measurement location, the type of sensor, the reference and tool, individual physiological attributes, and the surrounding environmental conditions. Our research underscores the critical need for further studies in this area to refine our understanding of these influential factors and to develop standardized guidelines for the use of NCITs and IRT.

## 1. Introduction

Body temperature is a vital sign that reflects a person’s health status. The regulation of body temperature is controlled by the hypothalamus, which uses the nervous system and body fluid to achieve heat production and heat dissipation in order to maintain the dynamic balance of body temperature [1,2]. Maintaining the appropriate body temperature level is crucial for the human body and normally ranges between 36.16 °C and 37.02 °C for healthy individuals [3]. Changes in body temperature can indicate the occurrence of different diseases. This change is a response to stress on the nervous and immune systems to pathogens, such as bacteria or viruses [4].

The importance of body temperature monitoring has been recognized since ancient times, with physicians in the early Roman Empire noting a relationship between body temperature and poor health [5]. The history of temperature measurement dates back centuries, with the development of various devices and techniques to monitor body temperature. Galileo Galilei invented one of the earliest thermometers in the late 16th century, and the mercury-in-glass thermometer was developed by Gabriel Fahrenheit in the early 18th century [6,7]. These early devices laid the foundation for modern temperature measurement techniques.

However, traditional contact thermometers, such as mercury or electronic thermometers, have limitations that can impact the accuracy and safety of temperature monitoring. For example, contact thermometers can affect the temperature field of the object itself when in touch with the object, resulting in potential temperature measurement errors [8]. In addition, contact methods require physical contact with the person being monitored, which can increase the risk of cross-infection and pose practical challenges for large-scale monitoring efforts [9,10].

The development of infrared technology in the mid-20th century introduced new possibilities for non-contact temperature sensing. This technology relies on the detection of infrared radiation emitted by objects to determine their temperature. Non-contact infrared thermometers (NCITs) and thermal scanners use this principle to measure the temperature of the skin surface without coming into contact with the skin. This method of temperature measurement can provide an accurate reading within seconds and it can be performed at a distance from several centimeters to meters away, reducing the risk of infection transmission [9]. Non-contact thermal screening, including the application of infrared thermography (IRT) and non-contact infrared thermometers (NCITs), is deemed a safe tool for temperature screening in the outbreak of infectious diseases.

Since the outbreak of COVID-19, non-contact temperature screening methods have been widely used in crowded places such as airports, markets, stations, and hospitals [11]. The pandemic has highlighted the need for rapid and accurate fever detection to mitigate the spread of the virus. This has led to an increased demand for non-contact temperature measurement devices and a surge in research evaluating their performance.

While non-contact infrared thermometers and thermal scanners have gained widespread popularity in recent years, their accuracy and reliability for detecting fever in humans are still a subject of debate. Several studies have suggested that non-contact tools are a reliable method with convenience and efficiency [9,10,11]. However, others have suggested that the accuracy and robustness of this method is not satisfying clinically and that contact sensors remain the preferred method of measurement.

To address this issue, we conducted a systematic review of the relevant literature to evaluate the quantitative accuracy and reliability of non-contact infrared thermometers and thermal scanners compared to reference standard tools, such as mercury thermometers, which often consist of contact-based methods. Our review aims to synthesize the available evidence on the performance of these devices and to provide a critical appraisal of the studies that have investigated their use for body temperature measurement and fever screening. By conducting this systematic review, we hope to provide guidance for appropriate sensor selection and future research in this area, as well as inform policy decisions related to the use of either contact or non-contact body temperature measurement methods.

## 2. Materials and Methods

### 2.1. Search Strategy

The structure of the content below follows the Preferred Reporting Items for Systematic Reviews and Meta-Analyses (PRISMA) guidelines [12]. A systematic literature search was conducted in multiple databases, including the Web of Science Core Collection, PubMed, and IEEE Xplore, to identify studies published before 31 March 2023 that reported the use of non-contact infrared body temperature measurement methods. The following keywords and search terms were used:

(Core Temperature OR Body Temperature) AND (measure* OR predi* OR monit* OR estimat*) AND (remote* OR camera* OR wireless* OR non-contact* OR non contact*)

The search was conducted by two independent reviewers, who screened the titles and abstracts of the identified studies to assess their relevance. Data were extracted from the eligible studies using a standardized form. The quality of the included studies was assessed using the Cochrane Risk of Bias tool.

### 2.2. Study Selection Criteria

We included studies that met the following criteria:

1. Comparison of non-contact infrared body temperature measurement with an established reliable measurement reference for body temperature;
2. Study conducted on human participants of any age, gender, or ethnicity;
3. Reporting quantitative measurements of body temperature;
4. Published in peer-reviewed journals;
5. Written in English.

Papers that did not meet these criteria we excluded. For this review, non-contact temperature measurement devices were defined as infrared thermometers, infrared thermal scanners, and infrared thermal cameras. The reference temperature was defined as the temperature obtained by other body temperature measurement sensors (which might also be a non-contact tool itself).

### 2.3. Data Extraction

Two independent reviewers extracted data from each included study using a standardized data extraction form. The following data were extracted:

1. Basic information of the study: author, year of publication.
2. Study methodology: place of experiment, type and brand of non-contact device used, environment temperature (mean environment temperature ± standard deviation of temperature, or the range of environment temperature), test distance, and measurement body site.
3. Population characteristics: group size, age structure, gender ratio.
4. Reference temperature: type and brand of reference tool used, measurement location on the body of the reference temperature (“body site”), and the distribution of the reference temperature (mean temperature ± standard deviation of temperature).
5. Quantitative results: measured temperature range, correlation coefficient between temperature obtained by non-contact devices and reference temperature, the mean difference (MD) of results between non-contact tools and reference tools, the standard deviation (SD) of the MD, and any other reported metrics of accuracy or reliability.

The mean difference (MD) is defined as [13]:

(1) $$MD=\frac{\sum_{i=1}^{n}\left(T_{dev,i}-T_{ref,i}\right)}{n}$$

where i is the index number of a single measurement; n is the total number of the measurements; $T_{dev,i}$ is the recorded temperature using the non-contact device from the ith measurement; $T_{ref,i}$ is the recorded temperature using the specific reference tool from the ith measurement.

The standard deviation (SD) of the MD is defined as:

(2) $$SD=\sqrt[{}]{\frac{\sum_{i=1}^{n}{\left[(T_{dev,i}-T_{ref,i})-MD\right]}^{2}}{n-1}}$$

where i is the index number of a single measurement; n is the total number of the measurements; $T_{dev,i}$ is the recorded temperature using the non-contact device from the ith measurement; $T_{ref,i}$ is the recorded temperature using the specific reference tool from the ith measurement; MD is the mean difference between non-contact tools and reference tools as calculated in Equation (1).

The full standardized data extraction forms are provided in Supplementary Tables S1 and S2.

Included studies might contain experiments with more than one non-contact device or reference tool. Each reported pair of non-contact device and reference tool was regarded as a separate test and thus one study might have multiple entries within the generated table.

The mean difference (bias) and the standard deviation (SD) between non-contact and reference systems were considered to be the primary outcome for the qualitative assessment of the included studies. If the mean difference was not reported in the study, the mean absolute error (MAE) or root mean square error (RMSE) was reported, but these studies were not taken into account for the quantitative analysis. The Supplementary Tables S1 and S2 also include any alternative performance measures that are applied in, e.g., fever screening, such as the sensitivity, specificity, area under the curve (AUC), positive prediction value (PPV), and negative prediction value (NPV).

### 2.4. Quality Assessment

The Quality Assessment of Diagnostic Accuracy Studies (QUADAS-2) tool was used to assess the quality of the included studies [14]. The quality assessment was performed by two independent reviewers, and any discrepancies were resolved through discussion.

### 2.5. Data Synthesis

We conducted a narrative synthesis of the available evidence due to the anticipated heterogeneity of the included studies. We summarized the characteristics and findings of each study and identified any patterns or inconsistencies in the results.

By following these methods, we aim to provide a comprehensive and transparent review of the available evidence on the comparison of non-contact infrared body temperature measurement with contact body temperature sensing.

## 3. Results

**Figure 1:**
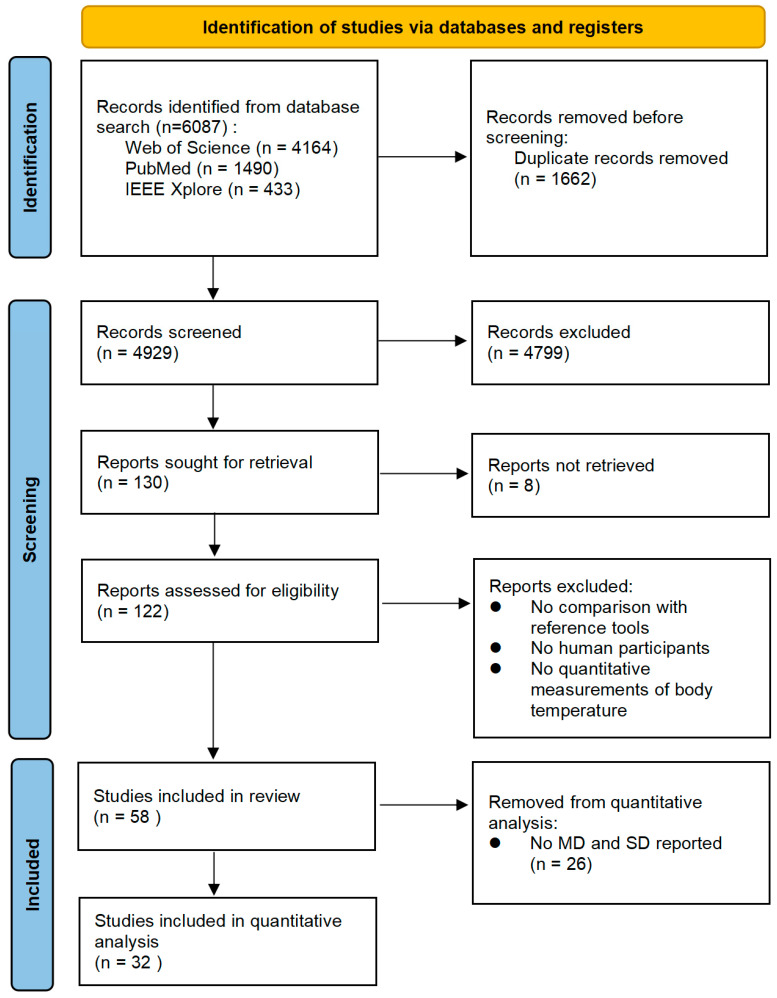
Preferred Reporting Items for Systematic Reviews and Meta-Analysis (PRISMA) flow diagram of the study selection process.

A total of 6087 articles were identified through the database search, of which 1662 were duplicates. After screening the titles and abstracts of the remaining 4929 articles, 130 records were selected for full-text screening. However, not all articles were obtainable from the University library system, online resources, or by contacting corresponding authors. A total of 122 records out of these 130 records were assessed based on their full text, as no access could be obtained for 6 identified manuscripts. Finally, 32 studies were included in the quantitative analysis of the systematic review (see Figure 1), as 26 studies had no reported MD or SD.

### 3.1. Characteristics of Included Studies

A total of 32 studies were included in the systematic review after screening 4929 records and assessing 122 full-text articles. The studies were conducted in various scenarios, including hospitals, rooms, and laboratory settings. These 32 studies contain 72 different experiment settings. The sample sizes ranged from a single person to 1113 participants, and the studies were conducted in different countries around the world. A total of 20 studies reported the range of their environment temperature, which varied from 9 °C to 38.8 °C. The included studies contain two non-contact body temperature devices: the non-contact infrared thermometers (NCITs) and infrared scanners, which use infrared thermography (IRT). A total of 21 studies (50 settings) used NCIT, and 13 studies (conducted in 22 settings) used IRT. A total of 28 studies reported the number of participants by gender. Among the 6792 participants of these 28 studies, 3426 (50.4%) were men and 3366 (49.6%) were women.

#### 3.1.1. Sensor Types

**Figure 2:**
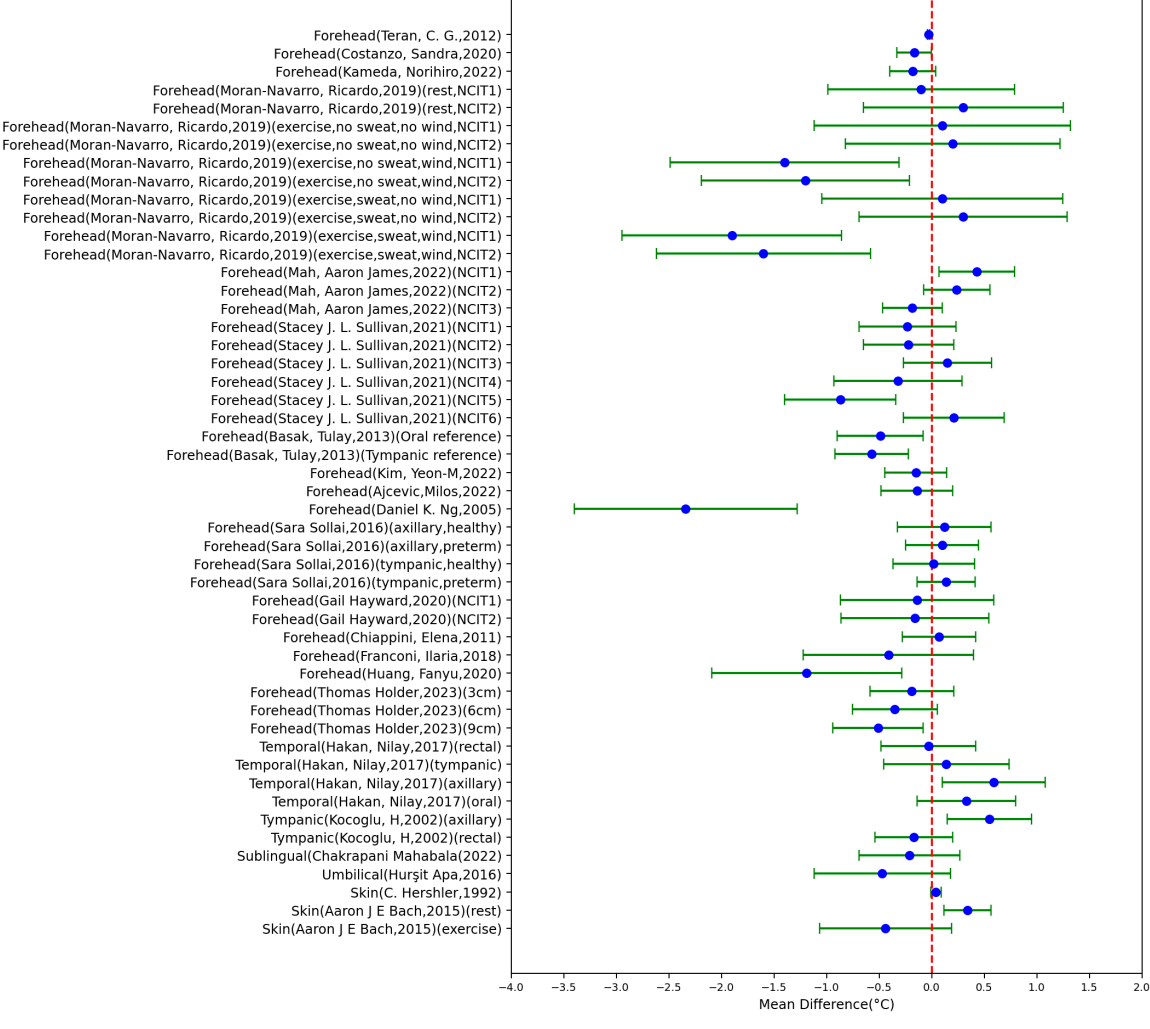
Forest plot comparing MD ± standard deviation of included studies of NCITs [13,15,16,17,18,19,20,21,22,23,24,25,26,27,28,29,30,31,32,33,34,35].

Table 1 summarize the characteristics of the included 21 papers using NCITs for body temperature measurement (50 settings), whilst Figure 2 depicts the MD ± SD of these studies. The measurement body site varied from forehead to fingers. The results showed that NCITs had varying levels of accuracy and reliability compared to contact-based methods. The highest absolute value of the MD was 2.34 °C (SD = 1.06 °C), which was measured from the forehead location of children from 1 month to 18 years old and compared with the tympanic thermometer. The lowest absolute value of the MD was 0.02 °C (SD = 0.388 °C), which was measured from the forehead location of healthy newborns and compared with a tympanic thermometer. A total of 21 settings out of 50 settings had an MD > 0 °C, while the other 29 settings had an MD < 0 °C.

**Figure 3:**
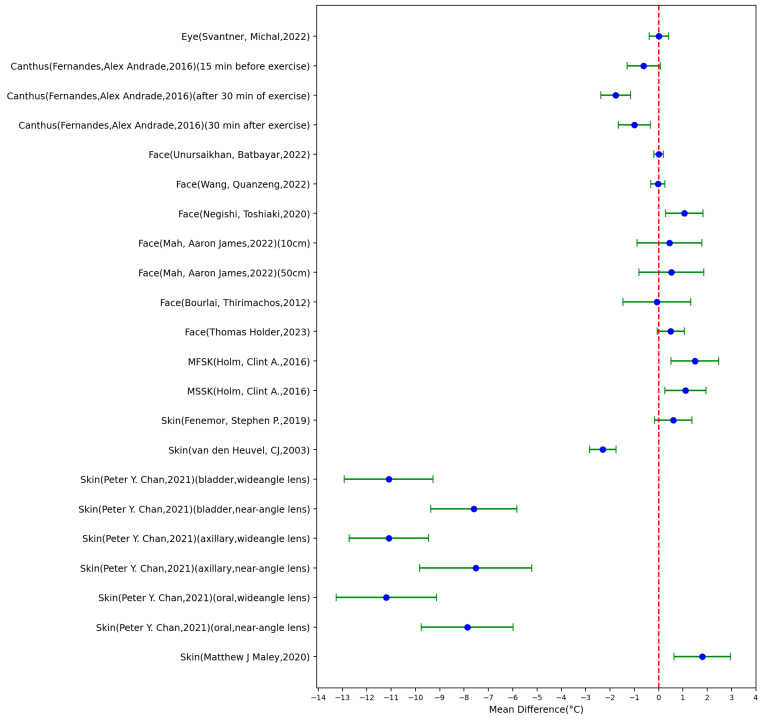
Forest plot comparing MD ± standard deviation of included studies of IRT [26,34,36,37,38,39,40,41,42,43,44,45,46].

Table 2 summarizes the characteristics of the included 13 studies that used infrared thermography (IRT) for body temperature measurement (22 settings), with Figure 3 showing the MD ± SD of these studies. The highest absolute value of the MD was 11.2 °C (SD = 2.056 °C), which was measured from the body skin of 61 adults with a mean age (±standard deviation) of 67 ± 15.2 years and compared the recorded data with that of an oral probe. The lowest absolute value of the MD was 0 °C (SD = 0.2 °C), which was measured at the face for a group of 154 children with COVID-19 and 147 healthy children. The IRT data from this study were compared to a mercury axillary thermometer. Across all studies, 9 settings out of the 22 settings had an MD > 0 °C, 1 setting had an MD = 0 °C, and the other 12 settings had an MD < 0 °C.

#### 3.1.2. Reference Temperature

The reference temperature utilized for comparison in the included studies varied, with measurements taken at different body locations, such as tympanic, axillary, oral, skin, rectal, and gastrointestinal sites. The selection of the reference temperature could have influenced the evaluation of the accuracy and reliability of the non-contact infrared temperature measurement methods.

**Figure 4:**
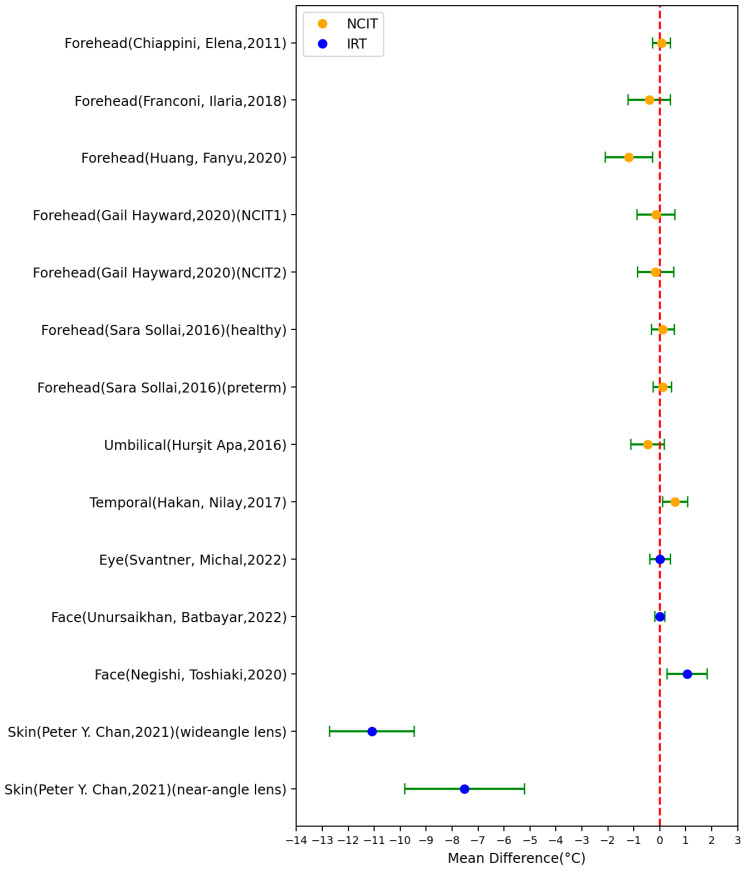
Forest plot comparing mean difference ± standard deviation (SD) of included studies with axillary temperature as reference [25,26,27,28,29,31,34,37,39,40].

Among the included studies, 11 studies (comprising of 14 settings) used axillary temperature as the reference body site, employing both digital or mercury thermometers (Figure 4). Out of these 14 settings, 6 had an MD > 0 °C, 1 had an MD = 0 °C, and the remaining 7 had an MD < 0 °C. The IRT study conducted by Unursaikhan Batbayar demonstrated the best MD and SD [37]. This study was conducted at the Central Hospital of Songinokhairkhan District in Mongolia; although the environmental temperature was not reported, the experiment was carried out under stable clinical conditions. The study included a group size larger than 100, with both male and female participants. The region of interest for body temperature was extracted using camera image processing algorithms. Conversely, the two settings of the IRT study conducted by Peter Y. Chan exhibited the worst MD and SD [45]. In this study, the software correcting a fixed offset was not used intentionally, resulting in a deviation greater than 7 °C.

**Figure 5:**
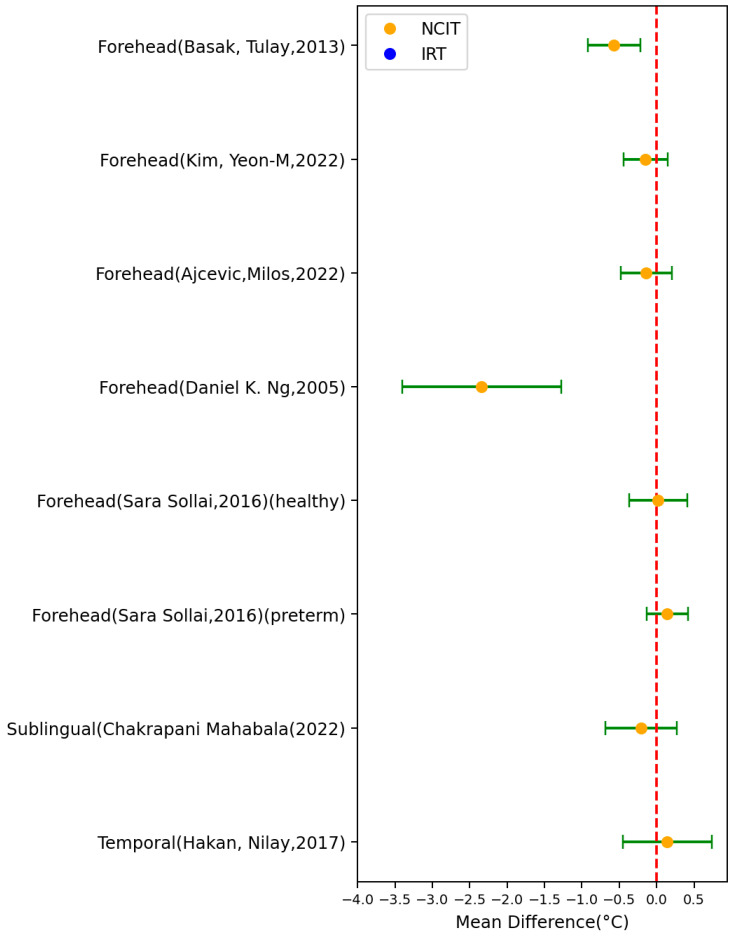
Forest plot comparing mean difference ± standard deviation (SD) of included studies with tympanic temperature as reference [21,22,23,24,25,31,33].

In seven studies (comprising eight settings), the reference temperature was measured using tympanic thermometers (Figure 5). Among these eight settings, three had an MD > 0 °C and five had an MD < 0 °C. One setting of the NCIT study conducted by Sara Sollai had the best MD and SD. This particular setting involved 70 pre-term newborns (28 boys and 42 girls) and was carried out in incubators [35]. On the other hand, the NCIT study conducted by Daniel K. Ng had the worst MD and SD [29]. This study was conducted at Kwong Wah Hospital and included 567 patients aged between 1 month and 18 years old.

**Figure 6:**
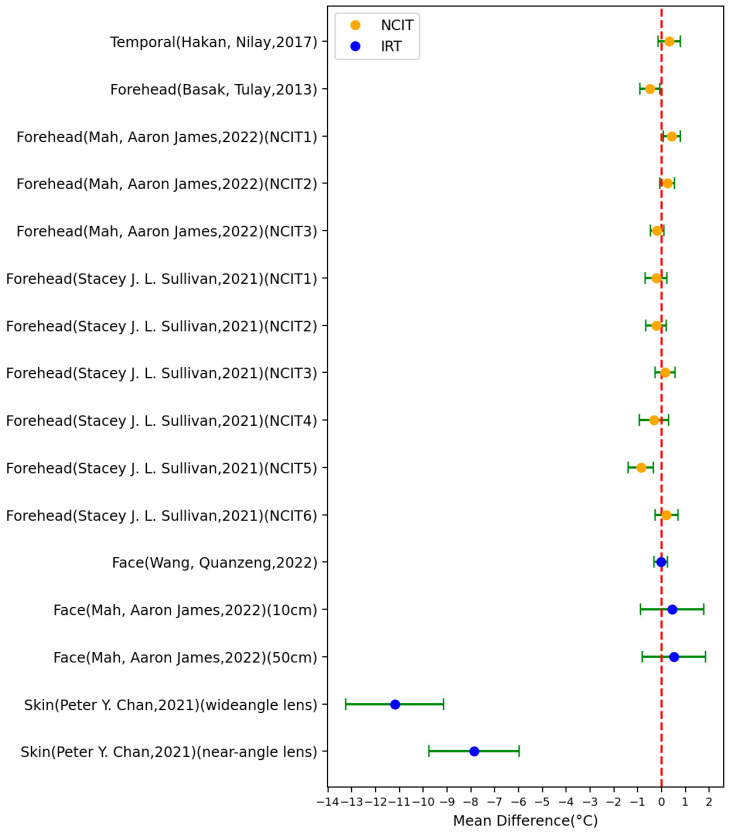
Forest plot comparing mean difference ± standard deviation (SD) of included studies with oral temperature as reference [19,20,21,31,41,46].

Regarding oral reference temperature measurements, 7 studies (covering 16 settings) used the oral cavity, employing various tools such as chemical dot thermometers, digital thermometers, mercury thermometers, or oral probes (Figure 6). Among these 16 settings, 7 had an MD > 0 °C and 9 had an MD < 0 °C. One setting of the IRT study conducted by Wang Quanzeng had the best MD and SD [38]. This study involved 1020 participants and was conducted at the Health Center of the University of Maryland. The ambient temperature ranged from 20 to 29 °C. The region of interest for body temperature in the IRT images was identified by matching landmarks on visible light images to thermal images using an image registration approach and manual labeling. Conversely, the two settings of the IRT study conducted by Peter Y. Chan exhibited the worst MD and SD [45]. These settings followed similar experimental conditions as the aforementioned axillary temperature findings reported in the same study.

**Figure 7:**
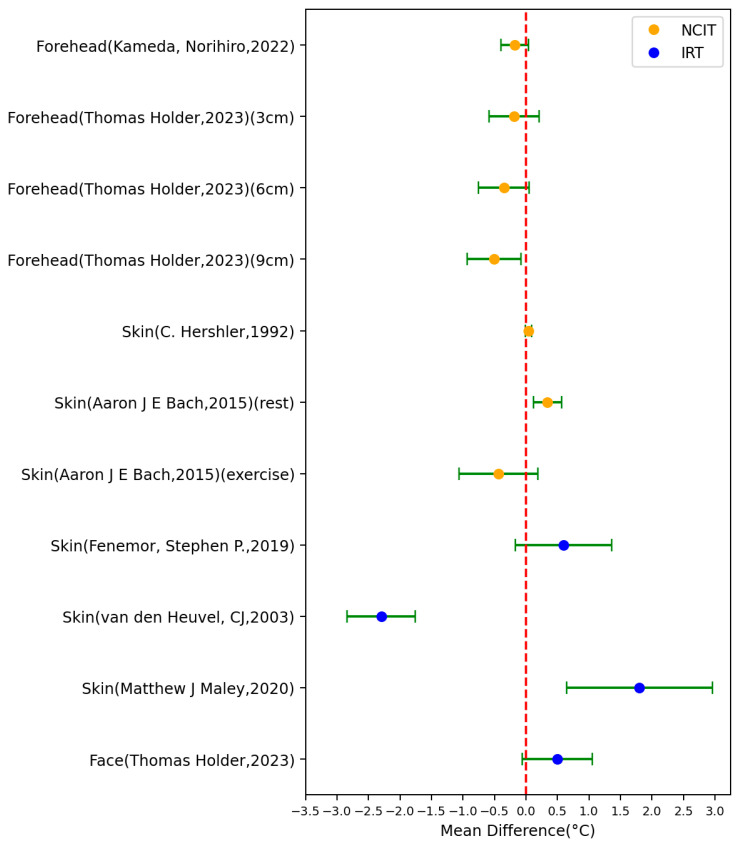
Forest plot comparing mean difference ± standard deviation (SD) of included studies with skin temperature as reference [14,17,30,35,36,44,45].

For skin temperature (non-axillary sensor location), 8 studies (comprising 11 settings) used this body site as the reference, employing tools such as zero-heat-flux cutaneous thermometers, thermistors, skin temperature probes, digital thermometers, and thermocouples (Figure 7). Among these 11 settings, 5 had an MD > 0 °C and 6 had an MD < 0 °C. One setting of the NCIT study conducted by C. Hershler had the best MD and SD, involving five male adults. Conversely, the two settings of the IRT study conducted by Matthew J. Maley exhibited the worst MD and SD. This study involved 52 male adults and was conducted in a climate-controlled chamber with an environmental temperature of 30.3 ± 0.9 °C.

Additionally, three studies reported rectal temperature as the reference, using mercury thermometers and rectal probes. Four studies used gastrointestinal temperature, measured by ingestible sensors, as the reference temperature. One study utilized bladder temperature measured via the bladder catheter.

The choice of reference temperature, as well as the calibration and maintenance of non-contact temperature measurement devices, are crucial factors to consider when assessing the accuracy and reliability of NCIT and IRT devices. These factors can directly impact the performance of non-contact temperature measurement methods in various clinical and non-clinical settings.

#### 3.1.3. Environment Temperature, Average Body Temperature, and Average Age

**Figure 8:**
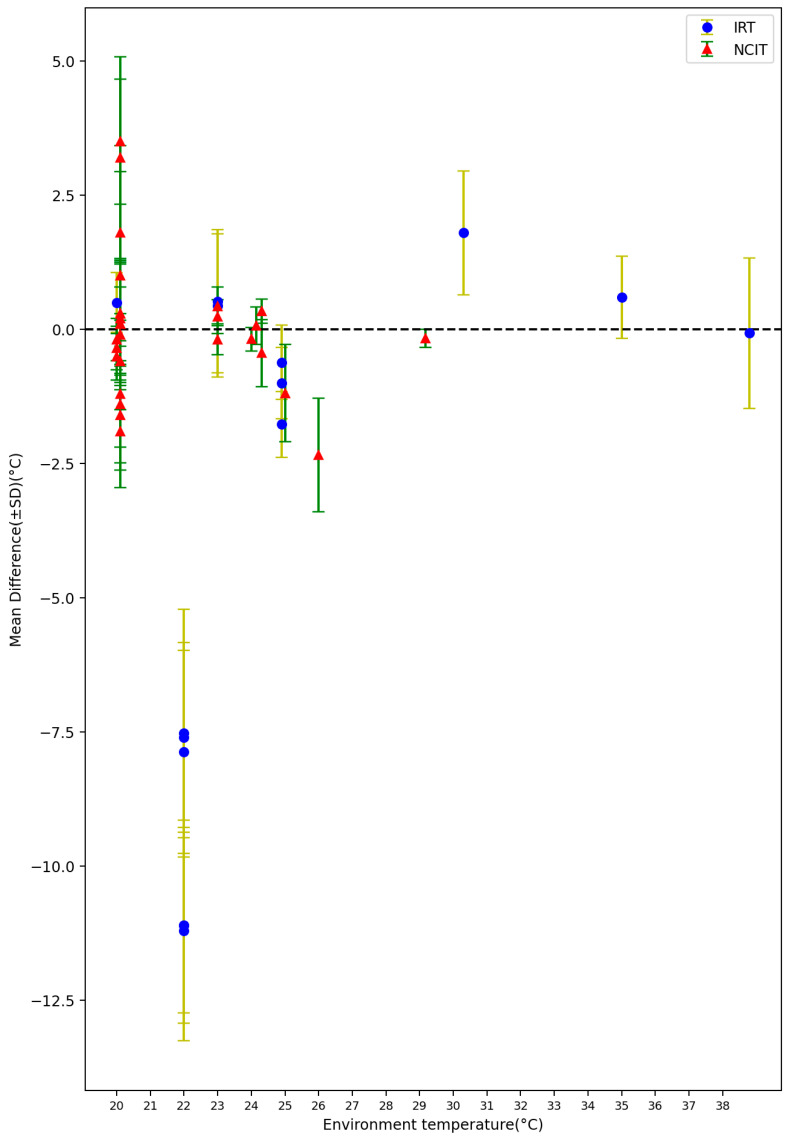
Forest plot comparing mean difference ± standard deviation (SD) of included studies with different environment temperature [14,16,17,18,19,24,27,29,30,36,38,42,44,46].

**Figure 9:**
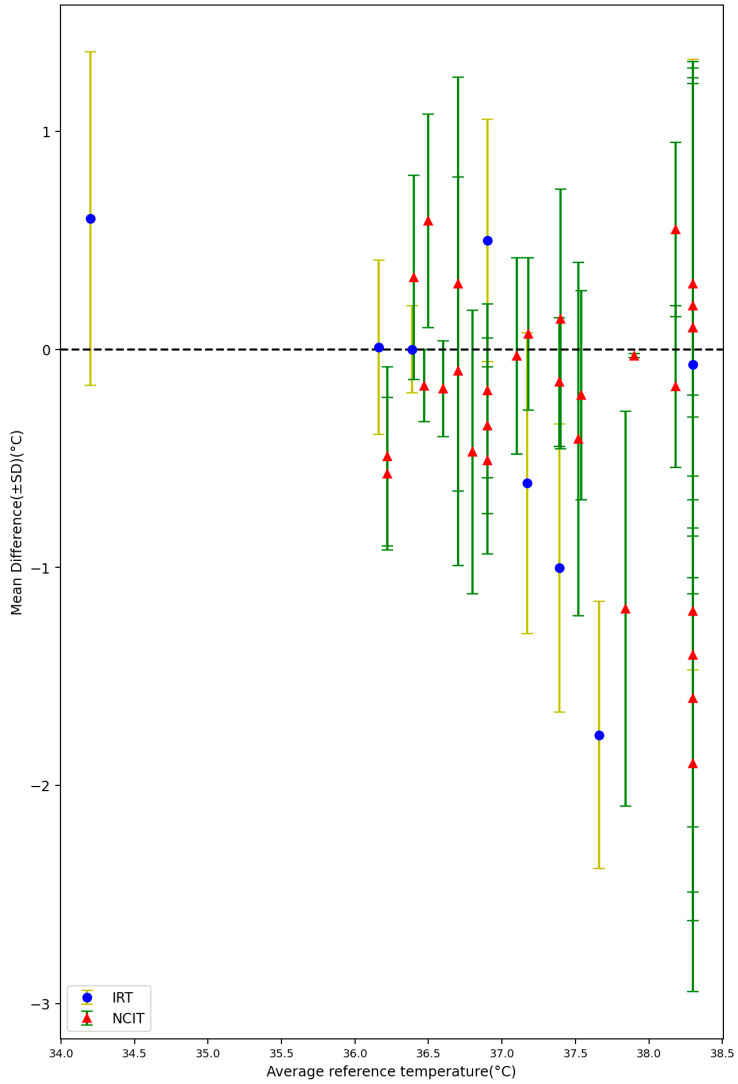
Forest plot comparing mean difference ± standard deviation (SD) of included studies with different average body temperature measured by reference tools [15,16,17,18,21,22,27,28,29,30,31,32,33,34,37,38,39,42,44].

**Figure 10:**
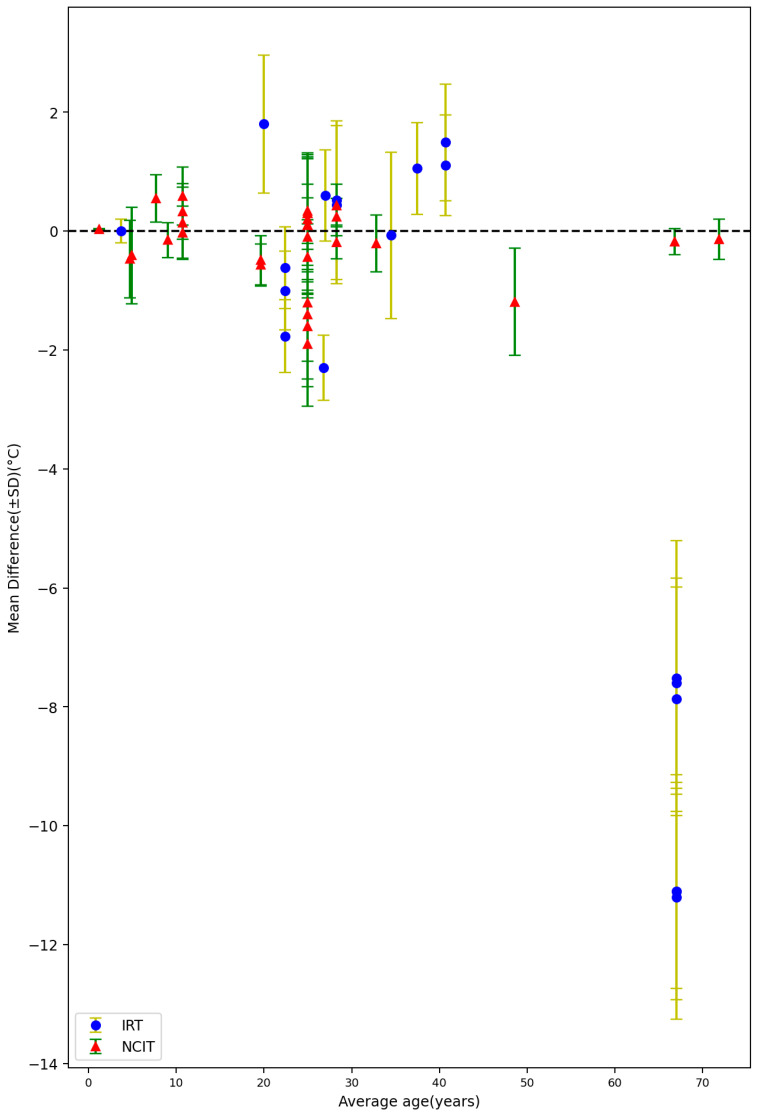
Forest plot comparing mean difference ± standard deviation (SD) of included studies with different average age of subjects measured by reference tools [14,15,17,18,19,21,22,23,28,29,31,32,33,34,36,38,39,40,42,43,44,46].

Among all of the 72 settings from the included 32 studies, 43 settings reported the average environment temperature varied from 20.1 °C to 38.8 °C. Figure 8 shows the mean difference between non-contact measurements and reference data against environmental temperature. A total of 38 settings reported the average reference temperature varied from 34.2 °C to 38.3 °C. Figure 9 shows the mean difference between non-contact and reference data when plotted against the average body temperature as obtained by the reference sensor. The lowest average body temperature (34.2 ± 1.0 °C) was reported by Fenemor, Stephen P [42], and Moran-Navarro, Ricardo reported the highest average body temperature, with a value of 38.3 ± 0.9 °C [23]. A total of 24 settings reported the average age of the subjects varied from 14.6 months to 67 years. Figure 10 depicts the mean difference between the non-contact devices and reference tools in relation to the subjects’ average age.

### 3.2. Methodological Quality of Included Studies

The Quality Assessment of Diagnostic Accuracy Studies (QUADAS-2) tool was used to assess the quality of all the included studies [47]. The risk of bias was assessed across four key domains, including patient selection, index test, reference standard, as well as flow and timing. Meanwhile, the patient selection, index test, and reference standard were also assessed for concerns regarding applicability. Supplementary Tables S1 and S2 summarize the results of the quality of the included studies.

Across the included studies, the risk of bias was generally low or unclear for patient selection and reference standard domains. However, the risk of bias was high or unclear for the index test domain in many studies due to issues such as lack of blinding or lack of clarity on the test threshold. Additionally, the applicability concerns were generally low or unclear for patient selection and index test domains, but high for the reference standard domain, due to variability in the reference standards used across the studies.

## 4. Discussion

Non-contact infrared thermometers (NCITs) and infrared thermography (IRT) have been widely recognized for their efficiency and safety in detecting body temperature, yielding to considerable benefits in healthcare settings. For example, by employing NCITs, nursing efficiency can be improved while minimizing patient discomfort and distress [48]. This review provides an overview of the performance of these devices.

This systematic review aimed to evaluate the accuracy and reliability of NCITs and IRT for human body temperature monitoring in various settings and conditions. A total of 32 studies were included, which demonstrated varying levels of accuracy and reliability for both NCIT and IRT devices when compared to reference standard tools, which were often based on contact sensing.

Our results show that the accuracy of NCITs is slightly better than that of IRT, although the gap is not very large. Generally, NCITs have a shorter effective distance and are less affected by external factors than IRT. Although, NCITs often exhibit higher accuracy and reliability compared to IRT, IRT still has its own unique advantages. IRT can be used to quickly screen the body temperature of large groups of people. For example, Armote Somboonkaew’s research shows that IRT can screen the body temperature of nine people simultaneously at a speed of eight frames/second [49]. A correlation coefficient of 0.731 was found between IRT and forehead skin thermometers. It achieves 100% sensitivity and 92.6% specificity under the cutoff value of 37.5 °C. Sun deployed thermal imagers at airports for body temperature screening, testing 617,289 individuals, with a mean difference of 2.7 °C [50].

The comparison results of different reference positions show that the measurement results of NCITs and IRT are not consistently higher or lower than those of a specific reference position. However, the wide range of mean differences indicate substantial variability in the performance of these devices. Factors such as the measurement body site, sensor type, reference standard, and individual differences may have contributed to these discrepancies.

The ISO 80601-2-56:2017 standard outlines the laboratory accuracy requirements for clinical thermometers [13]. Specifically, this accuracy is quantified as the measurement error, which is expressed as the mean difference in readings from a specific temperature source (such as a blackbody radiator or a fluid bath) as measured by both the clinical thermometer and the reference thermometer. For normal use, this measurement error should not exceed ±0.3 °C. However, the results from clinical studies reveal that this value is sometimes challenging to meet in practice with non-contact sensors. Among all of the 50 NCIT settings and the 22 IRT settings from the 32 articles included in our study, 29 NCIT settings and only 4 IRT settings managed to reach a mean difference within the range of ±0.3 °C.

The measurement accuracy of NCITs and IRT may be affected by various factors. First of all, the human body’s surface is not isothermal, meaning that temperatures can vary across different locations. If the non-contact device measures a different location than the reference device, discrepancies in temperature readings can occur due to the non-isothermal nature of the body’s surface. These variations can be influenced by factors such as blood flow, skin thickness, and underlying tissues at a particular measurement site [51]. Secondly, environmental factors, including temperature, humidity, wind speed, and nearby heat sources, can also impact the temperature of the body’s surface, thereby affecting measurement accuracy. Normally, the far-infrared emissivity of the body skin is 0.98. In the infrared range, skin emissivity decreases with increasing humidity [52]. The study of Moran-Navarro, Ricardo revealed that the MD of NCITs could reach −1.9 °C in a windy environment, while the MD of the same NCIT was only 0.1 °C without wind [18]. Thirdly, the operation distance of the NCIT and IRT can also influence the accuracy of body temperature. The accuracy tends to diminish as the distance increases [23,30]. Additionally, certain characteristics of the human body’s surface, such as skin color, perspiration, hair, and clothing, may influence the body’s heat radiation. These factors can further complicate the measurement process, creating variability in readings and making it more challenging to obtain consistent accurate outcomes [53,54]. Finally, differences between equipment makes and models can impact the accuracy and consistency of the outputs.

It is worth noting that different reference body sites and reference tools themselves have varying levels of accuracy and reliability. In this systematic review, our results show that the tympanic, axillary, skin, and oral temperatures are the most commonly used reference temperatures. Similar to non-contact measurement devices, the accuracy and reliability of reference tools are often limited by the manufacturer’s design and manufacturing process. A difference between the measured temperature of the reference body site and the true core body temperature of humans is likely to exist. Studies have shown that the rectum and gastrointestinal tract are closer to the real core body temperature of humans than other body sites. They are also less susceptible to external factors. Indeed, while rectal, gastrointestinal, and esophageal temperatures can be more invasive and challenging to measure, they are often considered the gold standard for body temperature measurement in clinical settings [55,56]. In determining a reference site for non-contact body temperature measurement, the balance between the convenience of selecting a certain location and the measurement accuracy is important.

Our results reveal that non-contact body temperature measurements can be conducted across various body positions, but the accuracy of these measurements can vary widely depending on the reference positions used. Among the studies we selected, the data were too heterogeneous to allow for a consolidated summary or the establishment of a general rule of thumb regarding the relationship between the body position measured by a non-contact thermometer and its accuracy. This highlights a significant gap in our understanding of how measurement and reference locations interact to influence accuracy. Further research is essential to explore these relationships more thoroughly, as a more precise understanding could lead to standardized guidelines or best practices for non-contact temperature measurement. Furthermore, the angle and distance of the measurement device in relation to the measurement location can further influence the outcome.

Our results show that most of the current comparative experiments on NCITs or IRT focus on indoor tests or tests in a controlled environment, with the temperature ranging from 20 to 38 °C, and there are few tests in low-temperature environments, despite the fact that the average temperature in many countries is lower than 20 °C, such as the United Kingdom [57]. Matthew J. Maley conducted a study in a climate-controlled chamber and showed that infrared cameras over-estimate skin temperature during re-warming after cold exposure [14]. This suggests that the accuracy of non-contact temperature measurement devices may be affected by temperature fluctuations and other environmental factors. This highlights that further research is needed to assess the applicability of non-contact body temperature measurement devices in lower-temperature environments, as low ambient temperature can significantly impact the physiological characteristics of participants and the performance of non-contact thermometers. Meanwhile, we should also pay attention to the influence of conditions such as humidity and clothing on non-contact body temperature measurement. At present, there are few studies focused on these aspects. Most of the included studies did not report the environmental humidity or the clothing of the participants. We suggested that environmental variables should be strictly controlled and reported in future studies.

Additionally, by comparing the MD and SD of different experimental setups at various mean reference body temperatures, our results showed that the MD and SD tended to be somewhat larger at higher body temperatures. Higher body temperature is often associated with increased metabolic activity. Variations in physiological activity accompanying higher body temperatures may induce a greater variability in the measured parameters, resulting in larger MD and SD values [58,59].

Furthermore, our results suggested a potential relation between the MD and the average age of subjects, especially for NCITs. Despite variations in settings and devices, both younger groups (average age < 20 years) and elder groups (average age > 50 years) showed better accuracy levels than groups with an average age ranging from 20 to 50 years. However, An V. Nguyen demonstrated that the differences between readings of NCITs and the oral reference temperature for older people were larger than those obtained for younger people [60]. This divergence underscores the importance of continued research within this area. Further investigations should be conducted to elucidate the underlying factors responsible for these variations and to optimize the accuracy of non-contact thermometers across all age groups. This would not only aid in a clearer understanding of the age-related nuances of temperature readings, but also guide clinicians in making more informed decisions based on NCIT measurements.

Though most of the included studies reported the gender ratio, none of them reported the difference of the accuracy between genders. Women typically have greater surface mass and higher subcutaneous fat content, leading to different thermal responses from men [61]. The study conducted by Eduardo Borba Neves showed that women have a lower skin surface temperature than men [62]. To improve the accuracy of non-contact body temperature measurements, further studies are needed to explore in more detail the accuracy difference between genders. In addition, none of the studies included in our review specifically investigated the impact of operator experience and training on the accuracy and reliability of non-contact temperature measurement devices. This highlights a potential area for future research in understanding the importance of operator proficiency in utilizing these devices. Moreover, among the included studies, none assessed the long-term stability and consistency of non-contact temperature measurement devices. However, it is crucial to note that, without understanding their long-term performance, the reliability of these devices for continuous monitoring or repeated measurements over extended periods remains uncertain. There might be potential drifts in accuracy due to incorrect device calibration or device aging. Future studies should be aimed at evaluating the long-term consistency of these devices, especially if they are to be used for continuous or prolonged monitoring.

The findings from the quality assessment for this systematic review have implications for the overall conclusions that are drawn. Studies with a high risk of bias can introduce potential biases that affect the accuracy and reliability of the results. Notably, the lack of random patient selection in several studies is a potential concern. Random patient selection is crucial for rigorous research design, as it ensures the generalizability and representativeness of the findings to the broader population. The absence of random selection can introduce performance and detection bias, leading to either the over-estimation or under-estimation of the accuracy of non-contact temperature measurement devices. For example, compared to young people, the average body temperature of the elderly is relatively lower due to reduced physical activity and inefficient thermoregulatory mechanisms, so the accuracy of temperature measurement may not be the same as that for young people [63]. For large-population monitoring, more work needs to be performed to include disabled volunteers in the sample population, as it is known that, for certain disabilities, chilled extremities or lower cardiac output could influence skin temperature readings [64].

Considering these limitations, it is important to interpret the conclusions drawn from this review with caution. While NCITs and IRT offer non-invasive and efficient methods for body temperature monitoring, their performance can be influenced by various factors. To mitigate these challenges, protocols should be developed that account for environmental conditions, individual characteristics, and potential biases associated with non-contact temperature measurement devices. Selection of contact sensors might be preferred if clinical accuracy is required.

Regarding fever screening, the accuracy of both NCITs and IRT is greatly influenced by the chosen cutoff temperature. A study by Daniel K. Ng investigated the effect of gradually increasing the fever screening cutoff temperature of NCITs from 34.2 °C to 36.0 °C [24]. The study found that, as the cutoff temperature increased, the sensitivity of the screening test gradually decreased from 97.56% to 50.41%, while the specificity increased from 18.81% to 93.96%. A similar trend was observed in Eddie Y.-K. Ng’s study on IRT [65]. As the cutoff temperature increased from 30.8 °C to 37.0 °C, the sensitivity of the screening test gradually decreased from 100.0% to 30.2%, while the specificity increased from 0.0% to 98.7%. These findings suggest that the choice of cutoff temperature is a critical factor in the accuracy and reliability of non-contact temperature measurement devices for fever screening. In the context of the COVID-19 pandemic, many public places, such as airports and hospitals, are using non-contact temperature measurement devices for fever screening. However, the choice of the appropriate cutoff temperature is still a matter of debate, and it is important to carefully consider the trade-offs between sensitivity and specificity when choosing a cutoff temperature for fever screening. When screening for fever, high sensitivity is often required to ensure that individuals with the disease are not missed. However, high sensitivity may result in lower specificity, potentially causing false positives, and leading to unnecessary further testing or treatment. Additionally, it is worth noting that the optimal threshold for fever screening may vary due to clothing, the surrounding environment, and individual differences. Furthermore, it is also important to note that fever is not always present in individuals with COVID-19. A study by Pana, Bogdan C. tested the performance of NCITs in COVID-19 screening [66]. When the cutoff temperature was set to 37.3 °C, the sensitivity of PCR-positive patients of COVID-19 was only 9.43%. Apart from body temperature, other symptoms or factors, such as exposure history, are also necessary to be considered in screening protocols.

Accurate and timely measurement of body temperature is crucial in identifying and preventing heat-related illnesses. Existing contact temperature detection devices (oral, axillary, ear, temporal, and forehead) measure temperatures that differ significantly from rectal temperatures and are inadequate for assessing hyperthermia in individuals exercising outdoors in the heat [67]. NCITs and IRT have the potential to offer non-invasive and efficient methods for monitoring body temperature in high-temperature environments, where physical contact with individuals may be impractical or uncomfortable.

However, it is important to consider the limitations and challenges associated with non-contact temperature measurement devices in, e.g., heat illness prevention. Environmental factors, such as high ambient temperature, humidity, and exposure to direct sunlight, can influence the accuracy and reliability of temperature measurements. The performance of these devices may be affected by factors such as sweat, evaporation, and heat radiation from the body’s surface. Additionally, variations in skin characteristics, such as skin color and moisture levels, may introduce further complexities in temperature measurement accuracy.

## 5. Conclusions

This systematic review synthesized 72 different settings from 32 research articles and presents a comprehensive evaluation of non-contact infrared body temperature measurement methods compared to contact body temperature measurement tools. Our findings suggest that non-contact infrared temperature measurement devices, specifically NCITs and IRT, can offer accurate and reliable temperature readings in specific settings and conditions. A total of 29 NCIT settings and 4 IRT settings have the absolute value of a mean difference < 0.3 °C, which is equal to the laboratory accuracy requirements of the ISO 80601-2-56:2017 standard. However, the inconsistency in the level of accuracy underscores the need for more research in this area.

We also found that the selection of the reference temperature tool and body site, as well as the calibration non-contact devices, greatly impacted their performance. Therefore, we recommend future studies to focus on establishing standardized protocols for non-contact temperature measurement, including the selection of appropriate reference standards and proper device calibration.

Notably, the quality assessment of the included studies revealed a high or unclear risk of bias in many studies for the participant selection domain, raising concerns about the overall quality of existing evidence. Consequently, future research should aim to adhere to a rigorous methodology to minimize bias and maximize the applicability of the results.

In summary, while non-contact infrared temperature measurement methods have promising potential in various clinical and non-clinical settings, further high-quality, methodologically sound research is necessary to fully elucidate their accuracy, reliability, and clinical usefulness.

## Figures and Tables

**Table 1:** The characteristics of studies using NCITs for body temperature measurement.

Year	Author	Scenario	Tool Brand	Measurement Body Site	Measurement Distance	Group Size	Age (Years)	Male	Female	Environment Temperature (°C)	Reference Results Distribution (°C)	Reference Temperature	Reference Tool	Mean Difference (MD) /Bias (°C)	Standard Deviation (SD) (°C)
2012	Teran, C. G. [15]	hospital	Thermofocus, model 01500, TECNIMED, Varese, Italy	forehead		434	14.6 ± 10.7 months	208	226	24 to 28	37.9 ± 0.9	rectal	rectal glass mercury thermometer	0.029	0.01
2020	Costanzo, Sandra [16]	room		forehead		individual				29.17 ± 3.04 (range 24 to 34)	36.47 ± 0.202			−0.1667	0.1647
2022	Kameda, Norihiro [17]	hospital	TM-1621; A&D Medical	forehead		65	66.8 ± 6.4 (range 50 to 80)	65	0	24	36.6 ± 0.1	skin (core temperature)	zero-heat-flux cutaneous thermometer	−0.18	0.22
2019	Moran-Navarro, Ricardo [18]	room, rest	Medisana, Germany	forehead	5 cm	12	25 ± 3.9	12	0	20.1 ± 0.3	36.7 ± 0.8	Core (gastrointestinal)	ingestible sensor	−0.1	0.89
		room, exercise, no sweat, no wind									38.3 ± 0.9			0.1	1.22
		room, exercise, no sweat, wind									38.3 ± 0.9			−1.4	1.09
		room, exercise, sweat, no wind									38.3 ± 0.9			0.1	1.147
		room, exercise, sweat, wind									38.3 ± 0.9			−1.9	1.045
		room, rest	Tecnimed, Italy	forehead	6 cm						36.7 ± 0.8			0.3	0.95
		room, exercise, no sweat, no wind									38.3 ± 0.9			0.2	1.02
		room, exercise, no sweat, wind									38.3 ± 0.9			−1.2	0.99
		room, exercise, sweat, no wind									38.3 ± 0.9			0.3	0.99
		room, exercise, sweat, wind									38.3 ± 0.9			−1.6	1.02
2022	Mah, Aaron James [19]	room	SCT01	forehead	2 to 5 cm	30	28.3 ± 9.4	14	16	23		oral	oral temperature sensor, Welch-Allyn	0.429	0.359
			FDIR-V16											0.237	0.315
			70121											−0.184	0.283
2021	Stacey J. L. Sullivan [20]	hospital	NCIT1, brand unknown	forehead	0.5 inches to 6 inches	1113		445	668	range 20.2 to 29.3		oral	Welch Allyn oral thermometer (SureTemp Plus 690, Welch Allyn, San Diego, CA, USA)	−0.23	0.46
			NCIT2, brand unknown											−0.22	0.43
			NCIT3, brand unknown											0.15	0.42
			NCIT4, brand unknown											−0.32	0.61
			NCIT5, brand unknown											−0.87	0.53
			NCIT6, brand unknown											0.21	0.48
2013	Basak, Tulay [21]	room	Thermosense	forehead	5 cm	452	19.66 ± 0.94	203	249		36.22 ± 0.10	oral	chemical dot thermometer	−0.49	0.41
												tympanic	tympanic thermometer	−0.57	0.35
2022	Kim, Yeon-M [22]	hospital	Hubdic Thermofinder S2, HFS-710, Gyeonggi-do, Korea	forehead	2 to 3 cm	255	9.05 ± 5.39	140	115	25 to 27	37.39 ± 0.66	tympanic	infrared tympanic thermometer	−0.15	0.295
2022	Ajcevic, Milos [23]	hospital	HG01, Comelit	forehead	10 cm	30	71.9 ± 18.9 (range 22 to 97)	24	6			tympanic	infrared tympanic thermometer, TEGENIUS3, Tyco Healthcare	−0.14	0.34
2005	Daniel K. Ng [24]	hospital	Standard ST 8812 (Standard Instruments Co., Hong Kong SAR, China)	forehead	5 cm	567	range 1 month to 18 years	335	232	26	range 36.0 to 41.5	tympanic	infrared thermometer (FirstTempHGenius, Intelligent Medical Systems Inc., Carlsbad, CA, USA)	−2.34	1.06
2016	Sara Sollai [25]	hospital incubator hospital incubator	Thermofocus, model 0800; Tecnimed, Varese, Italy	forehead		119 healthy newborns	Mean gestational age: 39 weeks + 6 days	64	55			axillary	SANITAS Hans Dislage GmbH	0.12	0.444
						70 preterm newborns	Mean gestational age: 27 weeks + 3 days	28	42					0.10	0.346
						119 healthy newborns	Mean gestational age: 39 weeks + 6 days	64	55			tympanic	Braun ThermoScan PRO 4000	0.02	0.388
						70 preterm newborns	Mean gestational age: 27 weeks + 3 days	28	42					0.14	0.276
2020	Gail Hayward [26]	hospital	Thermofocus	forehead		401	median 1.6	203	198			axillary	digital axillary thermometer, Welch Allyn SureTemp	−0.14	0.729
			Firhealth											−0.16	0.704
2011	Chiappini, Elena [27]	hospital	Thermofocus	forehead		251	range 3.0 to 8.6	127	124	24.15 ± 1.81	37.18 ± 0.96	axillary	mercury-in-glass thermometer	0.07	0.35
2018	Franconi, Ilaria [28]	hospital	Hartmann Thermoval Duo Scan (Model 925082; Hartmann, Germany)	forehead		205	4.89 ± 3.86 (range 0 to 14)	110	95		37.52 ± 1.09	axillary	digital axillary thermometer	−0.41	0.81
2020	Huang, Fanyu [29]	hospital	HYLOGY MD-H26	forehead	5 cm	26	48.6 ± 17.5	14	12	25	37.84 ± 1.02	axillary	mercury-free thermometer	−1.1892	0.9049
2023	Thomas Holder [30]	room	the JXB-182 Infrared Forehead Thermometer (IR gun) (Berrcom, China)	forehead	3 cm	119	range 20 to 59	50	69	20	36.9 ± 0.37	skin (core temperature)	SpotOn (3M, Saint Paul, MN, USA)	−0.19	0.398
					6 cm									−0.35	0.403
					9 cm									−0.51	0.429
2017	Hakan, Nilay [31]	hospital	ThermoFlash^®^ LX-26	temporal	10 cm	927	10.73 ± 16.32 (range 0.01 to 82)	358	569		37.1 ± 0.5	rectal	rectal thermometer	−0.03	0.45
											37.4 ± 0.9	tympanic	tympanic thermometer	0.14	0.596
											36.5 ± 0.7	axillary	axillary thermometer	0.59	0.49
											36.4 ± 0.5	oral	oral thermometer	0.33	0.469
2002	Kocoglu, H [32]	hospital	Braun ThermoScan IRT	tympanic		110	7.7 ± 2.2 (range 5 to 10)	66	44		38.18 ± 1	rectal	mercury thermometer	0.55	0.4
2022	Chakrapani Mahabala [33]	hospital	Accu DIGIT F1- BPL (India)	sublingual (oral) site	1 cm	35	32.8 ± 11.57				37.54 ± 0.93	tympanic	T-clinic TherCom cartable device (Manufacturer: Innovatec Sensing and Communication, Alicante, Spain. Model no: SN 58021315)	−0.21	0.48
2016	Hurşit Apa [34]	hospital	ThermoFlash LX-26, Visiomed SAS France, Paris/France	umbilical site	1.5 cm	100	56.3 ± 50.2 months (range 1 to 168 months)	53	47	range 24 to26	36.8 ± 1.03	axillary	axillary digital thermometer (Microlife MT 3001, Microlife AG Swiss Corporation, Widnau/ Switzerland)	−0.47	0.65
1992	C. Hershler [35]	room	FirstTemp@ (Intelligent Medi- cal Systems Inc., 6339 Paseo de Lago, Carlsbad, CA 92009, USA, or Pennco, 6 South Hill Park, London NW3 2SB, UK)	skin	1 to 3 cm	5	range 29 to 39 years	5	0		range 27.3 to 34.5	skin	thermocouple contact sensor (Thermalert TH-5)	0.04	0.05
2015	Aaron J E Bach [36]	room, rest	VisioFocus 06400 Infrared Thermometer, Tecnimed Inc., Varese, Italy	skin	60 mm	30	25.0 ± 2.9	30	0	24.3 ± 2.5		skin	two conductive devices (thermistors, iButtons) (EU-UU-VL5–0 Thermistors, Grant Instruments, Cambridge, UK; and DS1922L-F50 iButtons, Maxim Intergrated, Sunnyvale, California, USA)	0.34	0.224
		room, exercise												−0.44	0.6275

**Table 2:** The characteristics of studies using IRT for body temperature measurement.

Year	Author	Scenario	Tool Brand	Measurement Body Site	Measurement Distance	Group Size	Age (Years)	Male	Female	Environment Temperature (°C)	Reference Results Distribution (°C)	Reference Temperature	Reference Tool	Mean Difference (MD) /Bias (°C)	Standard Deviation (SD) (°C)
2022	Svantner, Michal [37]	room	FLIR Lepton	eye	230 cm	50 to 100	range 18 to 65			20 to 30	36.16 ± 0.38	axillary	armpit thermometer (electronic thermometers Microlife MT850)	0.01	0.4
2016	Fernandes, Alex Andrade [38]	room, 15 min before exercise	FLIR^®^, T420	inner canthus of the eye	1 m	12	22.4 ± 3.3	12	0	24.9 ± 0.6	37.17 ± 0.25	gastrointestinal	thermal pill, HQ CorTemp ^®^ Inc. (Palmetto, FL, USA)	−0.613	0.689
		room, after 30 min of exercise									37.66 ± 0.38			−1.769	0.613
		room, 30 min after exercise									37.39 ± 0.33			−1.002	0.661
2022	Unursaikhan, Batbayar [39]	hospital	FLIR Lepton 2.5	face	40 cm	154 (COVID) + 147 (healthy)	44.8 ± 14.8 months (COVID-19)/44.7 ± 14.1 months (healthy)	87 (COVID-19) + 70 (healthy)	67 (COVID-19) + 77 (healthy)		36.41 ± 0.20 (COVID-19)/36.37 ± 0.22 (healthy) (average 36.39)	axillary	mercury thermometer	0	0.2
2020	Negishi, Toshiaki [40]	room	FLIR A315	face	1 m	22 (healthy) + 41 (flu)	23.4 (healthy)/45.0 (flu)					axillary	clinical thermometer (TERUMO electric thermometer C230, TERUMO Co., Tokyo, Japan)	1.055	0.77
2022	Wang, Quanzeng [41]	room	A325sc, FLIR Systems Inc., Nashua, NH, USA	face		1020	>18	606	414	20 to 29		oral	oral thermometer (SureTemp Plus 690)	−0.03	0.29
2022	Mah, Aaron James [19]	room	FLIR One	face	10 cm	30	28.3 ± 9.4	14	16	23		oral	oral temperature sensor, Welch-Allyn	0.443	1.333
					50 cm									0.522	1.334
2012	Bourlai, Thirimachos [42]	room	FLIR Systems	face	1.5 m	6	34.5 ± 9.1	6	0	38.8 ± 1.0	38.3 ± 0.7	gastrointestinal	CoreTemp, HQ Inc, Ingestible Core Body Temperature Sensor	−0.07	1.4
2023	Thomas Holder [30]	room	FLIR C3 Thermal Camera (IR camera) (FLIR, USA).	face	0.6 m	119	range 20 to 59	50	69	20	36.9 ± 0.37	skin (core)	SpotOn (3M, USA)	0.5	0.556
2016	Holm, Clint A. [43]	room	Fluke Corporation, Everett, Washington	maximum frontal skin (MFSK) temperature		10	40.7 ± 9.9	10	0			gastrointestinal (core)	Ingestible Core Body Temperature Sensor, HQ Inc., Palmetto, FL, USA	1.491	0.984
				maximum side skin (MSSK) temperature										1.103	0.847
2019	Fenemor, Stephen P. [44]	room	Infrared Cameras Incorporated	body skin	1 m	11	27 ± 6	5	6	35	34.2 ± 1.0	skin	Thermistor (data logger)	0.6	0.765
2003	van den Heuvel, CJ [45]	hospital	MMS Med2000 camera	body skin		4	26.8 (range 22 to 31)	1	3		range 23.8 to 35.1	skin	Skin thermistors (Steri-Probe type 499B	−2.3	0.545
2021	Peter Y. Chan [46]	hospital	Thermal Experts TE-Q1 narrow-angle camera	skin	2.2 m	61	67 ± 15.2	35	32	22		bladder	Covidien Mon-A-Therm bladder catheter (Dublin, Ireland)	−7.6	1.77
												axillary	Welch-Allyn Suretemp Plus non-invasive probe (New York, NY, USA)	−7.52	2.31
												oral	Welch-Allyn Suretemp Plus non-invasive probe (New York, NY, USA)	−7.87	1.89
			QE1PLUS wide-angle (Daejeon, Korea) camera									bladder	Covidien Mon-A-Therm bladder catheter (Dublin, Ireland)	−11.1	1.826
												axillary	Welch-Allyn Suretemp Plus non-invasive probe (New York, NY, USA)	−11.1	1.632
												oral	Welch-Allyn Suretemp Plus non-invasive probe (New York, NY, USA)	−11.2	2.056
2020	Matthew J Maley [14]	climate controlled chamber (room)	A320G, FLIR Systems	index finger skin temperature	1 m	52	20 ± 2	52	0	30.3 ± 0.9		index finger skin	skin thermistor, Type EUS-U, Grant Instruments, Cambridge, UK	1.8	1.158

## Supplementary Materials

The following supporting information can be downloaded at: https://www.mdpi.com/article/10.3390/s23177439/s1, Table S1: NCIT; Table S2: IRT. References [68,69,70,71,72,73,74,75,76,77,78,79,80,81,82,83,84,85,86] are cited in the Supplementary Materials.
